# Glanders-Farcy and Mallein

**Published:** 1893-12

**Authors:** Simon J. J. Harger

**Affiliations:** Professor of Anatomy and Zoötechnics, Veterinary Department, University of Pennsylvania


### GLANDERS-FARCY AND MALLEIN.

### A Clinical Lecture.

By Simon J. J. Harger, Professor of Anatomy and Zoötechnics, Veterinary Department, University of Pennsylvania.

Gentlemen :—Perhaps in the whole category of the contagious diseases of the horse there is none so insidious in its onset, and at times difficult of recognition, as that of glanders. Now, first of all, you will remember that the various diseases designated by the name of acute glanders, acute farcy, chronic farcy, and chronic glanders are one and the same thing. They are identical. The difference being that farcy manifests its lesions on the external integument, while glanders has its lesions in the internal organs and the respiratory mucous membranes. Glanders is found in a very large number of animals. You will find it in the horse, the ass, the mule, the dog, the cat, in sheep, in goats, wolves and lions. It is said not to have existed in pigs, although recently a few cases have been reported. Cattle and barnyard fowls are exempt. A case of glanders in cattle has never been known. As you know it is also found in the human being. In the mule and in the ass we seldom see chronic farcy or glanders; it nearly always assumes the acute form. In the dog it may be generalized, and in some instances, however, it may remain localized at the seat of inoculation; the ulcers may heal and the animal afterwards be perfectly healthy. In the human being likewise it is usually generalized and fatal, although in rare cases it has remained localized and the individual has in those cases made a supposed recovery. Whether the recovery is absolute or whether it is only on the skin with some internal lesions still existing, are questions which yet remain to be determined.

The aetiology of this disease I suppose you have already studied. The predisposing causes are anything that tend to weaken or debilitate the system. I may mention working in cold rain, or in cold weather, unhygienic stables, poor feeding, excessive work, and the like. The exciting cause is the bacillus malleus, one of a large category of germs which produce such a large number of diseases.

The symptoms of farcy and glanders are, when they are characteristic, very characteristic; when they are not characteristic, they may be very obscure and there may be some difficulty in making a diagnosis. We have, however, in farcy a certain number of cardinal symptoms which are *four* in number. The first one of these is the *farcy bud.* In this case we find a number of them on the inside of the hind leg, which are small inflammatory foci or nodes in the subcutaneous tissue, varying in size from a hazlenut or a pea up to a walnut. These are at first hard and shot-like, and as they increase in size there is a gradual softening and suppuration in their interior, as the result of the cell proliferation, until the farcy bud is converted into an abscess. The pus having formed, the skin commences to necrose, sloughs off and leaves an *ulcer* which varies in size from a half-dime piece up to that of a silver dollar. Here for instance is an ulcer almost healed which is very small. On the inside of the hind leg you see small ulcers which are just commencing to ulcerate. If the ulcer refuses to heal for a long time the skin around gradually sloughs away until it reaches quite a large size.

Now you will see also the peculiar form of this ulcer, that it is irregular, that it is not round as an ordinary ulcer, but that its borders are irregular, zigzag, and that its edges instead of sloping away from the ulcer, as they do ordinarily in non specific ulcer, are perpendicular, and sometimes they overhang a surface of the ulcer. The bottom of the ulcer is of a greyish red or purplish color, which is rough and uneven.

The third symptom is the *discharge.* The discharge is purulent, or what might be termed purulo-albuminous. The characteristics of the discharge are a thick, viscid, albuminous, transparent substance which rolls down over the skin below, to which it has no tendency to adhere, and looks somewhat like olive oil. If you open one of the buds with a knife the discharge which comes from the abscess, instead of staining the skin and hair as it runs down, scarcely leaves any marks on its track. For instance, here we have a discharge at that point and the hairs are soiled only in certain places, while in other places they are perfectly clean. This is known to be a characteristic of the pus, a peculiarity which you do not find in any other discharge.

If you put your hand on the inside of the thigh of this horse you can feel the inflamed lymphatics in the form of thin cords running up towards the inguinal region to the lymphatic glands of the groin. These cords may gradually disappear or they may ulcerate. The inguinal glands may share the same fate and become indurated and enlarged. If you will examine this horse you will find that they are hard, indurated and nodulated. The ulcers, at times, have this important peculiarity, which you must not forget ; in the course of a certain length of time they may heal : they may cicatrize and leave nothing but a star-shaped cicatrix, and you are led to believe that your patient has made a good recovery. The ulcers may cicatrize as rapidly as new ones form, and the whole area may be spotted here and there with cicatrices generally. However, this is a case that will deceive you. Although the integument may show the absence of any lesion, if, at the same time, the horse has any tubercules in his lungs, he is capable of transmitting the disease and of infecting other horses with which he is brought in contact.

There was a horse in this hospital some years ago affected in exactly a similar manner, and a large number of ulcers developed on one of his hind legs—ulcers which readily cicatrized, healed, disappeared, and the horse apparently recovered. However, that horse was turned out to pasture and not only did he infect other horses, but in the course of five or six weeks he developed into a beautiful case of glanders. Thus you may have apparent recovery from glanders; your animal may appear perfectly healthy; he may show no sign of any disease; still during all that time he is a source of infection and may continually affect horses which are brought in contact with him. The absence of visible symptoms is no positive indication of the absence of glanders. This is the form which is called *latent* or incipient glanders, so called because there are no apparent symptoms. We have a number of occasional premonitory symptoms, such as acute lymphangitis with swelling of the extremities (usually the hind), which may come on in twenty-four or forty-eight hours. Bleeding from the nose without any apparent cause is also suspicious. Orchitis, inflammation of the testicle, in the entire horse, which may come over night or in the course of twenty-four or thirty-six hours, is also suspicious. These, however, are merely secondary symptoms.

This horse which you see here, as I have already said, was brought here from a stable where glanders exists; a stable from which two other horses were here in the hospital a few weeks ago suffering from glanders—farcy. The symptoms were not very marked. There was a slight swelling on the members. There were two ulcers on the skin, but no other symptoms. The nasal septum and the submaxilliary glands were normal. The general condition of the horse was that of cachexia; besides that we had the history of glanders. From these facts a diagnosis of glanders was tolerably positive. However, in order to be more certain of the diagnosis, I injected this horse as well as the other two with mallein.

Mallein was first prepared in 1891 in the Bacteriological Laboratory of the Veterinary School at Dorpat by a prominent and enthusiastic investigator, Kalning. This investigator prepared his mallein as follows: He took 5gm. of a pure glanders bacillus culture, to which he added 20ccm. of sterilized distilled water. This was exposed to a temperature of 120 degrees C. for 20 minutes, and repeated four times during the succeeding forty-eight hours. It was then exposed for forty-eight hours to 39 degrees C. and filtered through a Pasteur filter with the aid of a vacuum pump. The filtrate, consisting of 12cm., was now exposed to a temperature of 120 degrees C. for 15 minutes and was then ready for injection. You will note, as we have already learned, the effect of excessive heat on the attenuation of the virus.

With this lymph he injected three horses, artificially inoculated with glanders, with 1cm. each; in 10, 12, and 13 hours, respectively, there was a rise in temperature of 2 degrees and 2.7 degrees C.; a healthy horse injected simultaneously showed no rise in temperature. The accuracy of the lymph was then demonstrated by a post-mortem. Unfortunately this investigator himself died of glanders a short time afterwards from accidental inoculation. In handling glanderous material you can never be too careful about your own person. Subsequently other investigators followed, among them was Preusse, the German investigator, and one of my colleagues, Dr. Pearson. Preusse's lymph was made from potato culture of the bacillus dissolved in equal parts of glycerine and water. It was used in the form of a solution, one part to ten parts of a two per cent. solution of carbolized water. With this material he injected guinea pigs as well as horses. He injected six horses which showed no characteristic symptoms, and the diagnosis was quite uncertain, with 1 to 3ccm. of the lymph, followed in 7½ hours by a second injection of 3 to 5ccm. In 5 of these, in the course of 15 hours, there was a rise in the bodily temperature of from 3 to 5 degrees C. The post-mortem proved the existence of glanders, whilst in the sixth, which was a foal two weeks old, there was no rise in temperature and no disease was found on post-mortem examinations.

Other investigators, among them Shilling and Dickerhoff, used the lymph in the same way in doses of from 1 to 3cm. on 112 horses. Of these 112 horses, 66 showed an elevation of the bodily temperature. Of these 66, 63 showed by a post-mortem that they were affected with glanders. The remaining three did not show any lesions of glanders, and in two of them no lesions at all could be found; in the third there was a slight tubercular deposit in the lungs, but the inoculation of a guinea pig produced a negative result. Still that is not positive in view of the fact that inoculation of the glanderous material in guinea pigs is not always positive, and an inactive inoculation is no positive proof of the absence of glanders. The temperature in all these cases reached its maximum in from nine to ten hours. There were also slight constitutional symptoms, as dullness, slight shivering, and a certain amount of inappetence. The remaining 46 showed no elevation in temperature, and on post-mortem were found to be perfectly healthy.

The most thorough investigation with mallein was made by the French investigator, Nocard. A strong solution of mallein was made by him in this way: A culture of the bacillus in peptonized glycerine bouillon (the same as for tuberculine) was exposed to a temperature of 35 degrees C. for one month. At the end of this time it was sterilized at a temperature of 110 degrees C. It was then filtered through paper and evaporated to one-tenth of its volume in a vacuum and in the presence of sulphuric acid. You will thus see that this preparation was a very strong lymph. It was used medically in a ten per cent. solution of carbolized water. The carbolized water was made of five parts of carbolic acid to one thousand parts of water, so as to diminish the irritating effects of this material. The dose of this lymph is about a quarter of a ccm. A large dose, one-half to one ccm., produced in a normal horse a slight inflammatory swelling, which disappeared in from one to two days, and a rise in temperature of from 1 to 2 degrees C. You will see that you have here a reaction in the healthy horse, but the dose is much too large. A quarter of a ccm. in a healthy horse produces no reaction, while in subjects with glanders or farcy it produces a decided reaction. Hence, here was the difference of the action on the healthy and on the diseased horse on which the diagnostic value of the material is based.

In this manner Nocard injected 48 horses; in 34 there was an elevation of temperature of from 2 to 3 degrees, and after death he found glanders. Eleven showed no results whatever, no fever and no disease. The remaining three were at first somewhat disappointing. There was respectively an elevation of temperature of 1 degree, 1.4 degrees and 1.8 degrees. The post-mortem examination, however, demonstrated that these animals suffered from bronchitis, strangles and melanosis, to which the slight rise of temperature was then attributed. Subsequently experiments were made on diseased animals, animals suffering from bronchitis, pleurisy, collection of the nasal sinuses, sarcoma of the jaw, etc., with negative results. There was in these cases no rise in temperature which could be mistaken for that which results from glanders or farcy.

In one case he experimented on a horse which had "enlarged gland" (but I could not find out what the enlargement was due to), and after three successive injections there was a rise in temperature of 1 degree. The submaxilliary gland was then removed and the horse again injected with no reaction; the presence of the diseased gland evidently in that case had been the cause of the slight rise in temperature.

Mallein contains an active principle to which, if not the entire, at least part of its activity is due. This active principle is precipitated by alcohol as a flocculent deposit, filtered, and then washed with sulphuric acid ; it then exists as a white crystalline alkaloid—it might be termed alkaloid—very soluble in water, in which it makes a slightly yellowish solution. As it is ordinarily prepared, mallein contains about 1½ per cent. of this alkaloid, for which as yet nobody has been ambitious enough to discover a name. This same alkaloid exists in the serum of the organs of horses that have died from glanders. Its physiological effects on the animal economy is the same as that of mallein. It is used in doses of 0.15gm.

The mallein of which I have here a sample, given to me by the Bureau of Animal Industry, is a slightly yellowish, syrupy, citron-colored fluid of a peculiar odor and of a neutral or slightly acid reaction. The method by which it was prepared I do not know; but in all cases the process is the same, the primary object being to obtain a solution of the ptomaine or poisonous principle that is excreted by the bacillus. This mallein when injected in doses of 1ccm. in a healthy horse produces but little effect. It may produce a slight local swelling, which passes off in a day or two, and an occasional rise of temperature of from ½ to 1 degree C. In the diseased animal it produces marked symptoms. In the first place it causes, at the point of inoculation, a large, hard, hot, limited inflammatory swelling which varies in size from a hen's egg to a child's head, which in the course of 48 hours gradually disappears, and may not show any trace after five or six days. The temperature may rise from 3 to 6 degrees F. If there is a rise of temperature of no more than 1 degree C., the animal is not diseased and you cannot make a diagnosis of glanders. A rise of temperature of between 1 and 2 degrees C. is suspicious, but not positive, and the animal must show a rise of temperature above 2 degrees C., or at least 103 F., in order to be of any positive value.

Before arriving at a definite conclusion, if the reaction is not decided, it is best to make in the course of 24 hours a second injection, and if necessary in 24 hours more a third injection, in order to demonstrate absolutely whether the fever is due to a diseased condition or merely to the activity of the lymph itself. The best time to make the injections is in the morning. When you inject a diseased horse it is well to inject a healthy horse also. The dose of this lymph is 1ccm.

I have here a record of its effect on those horses which I have injected. First of all, I will call your attention to the table showing the action of the lymph of a healthy horse.

### TABLE NO. I.

*Showing effects of mallein on a healthy horse.**

| | July 4th. | | | | | | July 5th. | | | | | |
|---|---|---|---|---|---|---|---|---|---|---|---|---|
| | 8 p.m. | 10 | 12 | 2 | 4 | 6 | 8 | 10 | 12 | 2 | 4 | 6 p.m. |
| Pulse, | 38 | 40 | 44 | 44 | 40 | 46 | 40 | 38 | 40 | 42 | 42 | 44 |
| Temperature, | 16 | 16 | 18 | 16 | 14 | 15 | 16 | 14 | 20 | 14 | 14 | 16 |
| Respiration, | 38.4 | 38.3 | 38.2 | 38.3 | 38.5 | 38.4 | 38.5 | 38.7 | 38.6 | 38.5 | 38.6 | 38.6 |

Injected at 8 p. m., July 4th, 1892, 1 gm. mallein. Second injection, 8 a. m., July 5th, 2 gm. mallein.

The first inoculation was made on the 4th day of July, 1892, the dose was 1 gm. mallein; the second on the 5th, at 8 A.M., two successive inoculations. The chart shows the state of the pulse and the number of respirations. You will notice the pulse is always uniform, never passing above 44, and you will also notice there is little or no alteration in the respiration and the temperature; no local alterations.

* Monatshelfte fur practische Thierheilkunde.

### TABLE NO. II.

| Before Injection. | | | When Injected. | | | After Injection. | | |
|---|---|---|---|---|---|---|---|---|
| Date. | Time of day. | Temperature. | Date. | Time of day. | Temperature. | Date. | Time of day. | Temperature. |
| Aug. 21, | 12 m. | 100.4 | 22 (Aug.) | 6 a.m. | 100. | Aug. 22 | 8 a. m. | 100. |
| " | 6 p.m. | 100.4 | 1 c. c. mallein. | | | " | 10 a.m. | 100. |
| " | 10 p.m. | 101. | .... | .... | .... | " | 12 m. | 100.9 |
| .... | .... | .... | .... | .... | .... | " | 2 p.m. | 102.4 |
| .... | .... | .... | .... | .... | .... | " | 4 p.m. | 104.2 |
| .... | .... | .... | .... | .... | .... | " | 6 p.m. | 106. |
| .... | .... | .... | .... | .... | .... | " | 8 p.m. | 105.8 |
| .... | .... | .... | .... | .... | .... | " | 10 p.m. | 105.4 |
| .... | .... | .... | .... | .... | .... | " | 12 p.m. | 104.7 |
| .... | .... | .... | .... | .... | .... | Aug. 23 | 6 a.m. | 104. |
| .... | .... | .... | .... | .... | .... | " | 9 a.m. | 104.6 |
| .... | .... | .... | .... | .... | .... | " | 10 a.m. | 104.1 |
| .... | .... | .... | .... | .... | .... | " | 12 m. | 104.2 |
| .... | .... | .... | .... | .... | .... | " | 2 p.m. | 103.6 |
| .... | .... | .... | .... | .... | .... | " | 4 p.m. | 103.8 |
| .... | .... | .... | .... | .... | .... | " | 6 p.m. | 104.3 |
| .... | .... | .... | .... | .... | .... | " | 8 p.m. | 105. |
| .... | .... | .... | .... | .... | .... | " | 10 p.m. | 104 6 |
| .... | .... | .... | .... | .... | .... | " | 12 p.m. | 103.6 |
| .... | .... | .... | .... | .... | .... | Aug. 24 | 8 a.m. | 102.8 |
| .... | .... | .... | .... | .... | .... | " | 12 m. | 103. |
| .... | .... | .... | .... | .... | .... | " | 2 p.m. | 103. |
| .... | .... | .... | .... | .... | .... | " | 4 p.m. | 103.2 |
| .... | .... | .... | .... | .... | .... | Aug. 25 | 8 a.m. | 101.9 |
| .... | .... | .... | .... | .... | .... | " | 12 m. | 101.6 |
| .... | .... | .... | .... | .... | .... | " | 4 p.m. | 101 9 |
| .... | .... | .... | .... | .... | .... | Aug. 26 | 8 a.m. | 100.1 |
| .... | .... | .... | .... | .... | .... | " | 12 m. | 100.2 |
| .... | .... | .... | .... | .... | .... | " | 4 p.m. | 101. |

### TABLE NO. III.

| Before Injection. | | | When Injected. | | | After Injection. | | |
|---|---|---|---|---|---|---|---|---|
| Date. | Time of day. | Temperature. | Date. | Time of day. | Temperature. | Date. | Time of day. | Temperature. |
| Aug. 22, | 12 m. | 100.9 | Aug. 23, | 6 a.m. | 100.2 | .... | .... | .... |
| " | 2 p.m. | 101. | 1 c. c. mallein | | | 23 (Aug.) | 9 a.m. | 101.6 |
| " | 4 p.m. | 101.2 | .... | .... | .... | " | 10 a.m. | 101.8 |
| " | 6 p.m. | 101.4 | .... | .... | .... | " | 12 m. | 102. |
| " | 8 p.m. | 101.7 | .... | .... | .... | " | 2 p.m. | 103.6 |
| " | 10 p.m. | 100.9 | .... | .... | .... | " | 4 p.m. | 104.8 |
| " | 12 p.m. | 101. | .... | .... | .... | " | 6 p.m. | 105. |
| .... | .... | .... | .... | .... | .... | " | 8 p.m. | 105. |
| .... | .... | .... | .... | .... | .... | " | 10 p.m. | 104.8 |
| .... | .... | .... | .... | .... | .... | " | 12 p.m. | 103.8 |
| .... | .... | .... | .... | .... | .... | Aug. 24 | 8 a.m. | 103. |
| .... | .... | .... | .... | .... | .... | " | 10 a.m. | 103.4 |
| .... | .... | .... | .... | .... | .... | " | 2 p.m. | 104. |
| .... | .... | .... | .... | .... | .... | " | 4 p.m. | 103.4 |
| .... | .... | .... | .... | .... | .... | Aug. 25 | 8 a.m. | 102. |
| .... | .... | .... | .... | .... | .... | " | 12 m. | 102.1 |
| .... | .... | .... | .... | .... | .... | " | 4 p.m. | 101.8 |
| .... | .... | .... | .... | .... | .... | Aug. 26 | 8 a.m. | 101.9 |
| .... | .... | .... | .... | .... | .... | " | 12 m. | 102. |
| .... | .... | .... | .... | .... | .... | " | 4 p.m. | 102. |

These cases were suspicious of farcy-glanders, but the symptoms were not marked.

The local symptoms were as follows:

After the injection a swelling developed at the seat on inoculation about the size of a hen's egg, and it increased a little up to ten P.M. On the 24th, at 8 A.M., it was twice as large as on the preceding day. On the 25th, at 12 M., it was smaller, and on the 26th was the size of a walnut and practically had disappeared.

### TABLE NO. IV.

| Befoie Injection. | | | When Injected. | | | After Injection. | | |
|---|---|---|---|---|---|---|---|---|
| Date. | Time of day. | Temperature. | Date. | Time of day. | Temperature. | Date. | Time of day. | Temperature. |
| Sept. 21, | 12 m. | 104.8 | .... | .... | .... | ... | ... | .... |
| Sept. 22, | " | 102. | Sept. 23, | 9 a.m. | 102.2 | Sept. 23, | 10 a.m. | 102.2 |
| .... | .... | .... | 1 c. c. | .... | .... | " | 12 m | 102.2 |
| .... | .... | .... | .... | ... | .... | " | 2 p.m. | 101.4 |
| .... | .... | .... | .... | .... | .... | " | 4 p.m. | 101. |
| .... | .... | .... | .... | . . | .... | " | 6 p.m. | 102. |
| .... | .... | .... | .... | .... | .... | " | 8 p.m. | 103.6 |
| ... | ... | .... | .... | ... | .... | " | 10 p.m. | 103.8 |
| .... | .... | .... | .... | .... | .... | " | 12 m. | 104.4 |
| .... | .... | .... | .... | .... | .... | Sept. 24 | 6 a.m. | 102 8 |
| .... | ... | .... | .... | .... | .... | " | 8 a.m. | 102.2 |
| .... | .... | .... | .... | . .. | .... | " | 10 a.m. | 102.2 |
| ... | .... | .... | .... | .... | .... | " | 12 m. | 102.2 |
| .... | .... | .... | .... | . | .... | " | 2 p.m. | 102. |
| .... | .... | .... | .... | .... | .... | " | 4 p.m. | 101.4 |
| ... | .... | ... | .... | .... | .... | " | 6 p.m. | 102.2 |

Now we have a third case, the one which we studied a minute ago, which is not as positive in its reaction from this lymph as the other two preceding, and we did not use the greatest precaution in this case. Firstly, for the reason that on September 21st, at 12 M., two days before the inoculation, the temperature was 104.8 degrees Fahr. On September 22d, at 12 M., the temperature was 102 degrees Fahr. Now, in order to be more accurate, the temperature of this horse should have been taken the next day, and the next day, until it had been uniform for a certain number of days in succession, in order to obtain the actual temperature prior to the inoculation, the actual temperature uninfluenced by accidental conditions. The animal was injected on the 23d, the bodily temperature being 102.2 degrees Fahr., at ten A.M., with 1 C. C. of mallein. The temperature was quite high for a chronic case. One hour afterwards the temperature was the same, 102.2 degrees. It was taken successively every two hours, and reached its maximum at 12 P.M., or 15 hours after the injection, when it was 104.4 degrees Fahr., only a rise of 2.2 degrees. This was a small rise, and had it been a case involving a great deal of importance and reputation another injection should have been made the next day, in order to ascertain whether there would be a greater elevation of temperature. In this case we did not consider it necessary, and I did not repeat the injection. The local symptoms were the same as in the other cases. The horse is now killed and the post mortem made, and although the action of the mallein was not as decisive as might have been. we now see that the diagnosis is correct, after a few tubercles in the lungs and lobular (triangular) pneumonia are found to exist.

I wish to say one word more as to the practical value of mallein under certain conditions. First, if you find farcy or glanders in a stable, such as is in the stable where this horse came from, you will destroy those absolutely diseased. It would be economy for the proprietor of such stable to employ a veterinarian, and have him inject, with mallein, every animal in the stable; and for this reason, as I said a while ago, that the animal may be in an apparently good condition, as far as one is able to see, and nothing may be detectable, at the same time that he may be the source of a serious and constant inoculation that may infect all the other animals in the stable. It is only possible in such cases to demonstrate the existence of the disease by one or more injections of this lymph. I know a stable in this city now where glanders exists, and where I saw a horse with absolutely characteristic ulcers on a hind pastern, which apparently had been cured, but I venture to say that an injection of mallein would successfully demonstrate the existence of latent glanders. I have collected 191 cases, which have been treated with mallein. The results of the injections were positive in all with the exception of the two which I have previously mentioned.

